# Litter size manipulation in laboratory mice: an example of how proteomic analysis can uncover new mechanisms underlying the cost of reproduction

**DOI:** 10.1186/1742-9994-11-41

**Published:** 2014-05-20

**Authors:** Marine I Plumel, Antoine Stier, Danièle Thiersé, Alain van Dorsselaer, François Criscuolo, Fabrice Bertile

**Affiliations:** 1 Département Sciences Analytiques, Institut Pluridisciplinaire Hubert Curien, CNRS UMR7178, 25 rue Becquerel, 67087 Strasbourg, Cedex 2, France; 2 Département d’Ecologie, Physiologie et Ethologie, Institut Pluridisciplinaire Hubert Curien, CNRS UMR7178, 23 rue Becquerel, 67087 Strasbourg, Cedex 2, France; 3 University of Strasbourg, 4 rue Blaise Pascal, F-67081 Strasbourg, Cedex, France

**Keywords:** Reproduction cost, Trade-off, Proteomics, Oxidative stress, Ageing, Mice

## Abstract

**Background:**

Life history theories predict that investment in current reproduction comes at a cost for future reproduction and survival. Oxidative stress is one of the best documented mechanisms underlying costs of reproduction to date. However, other, yet to be described molecular mechanisms that play a short term role during reproduction may explain the negative relationships underlying the cost of reproduction. To identify such new mechanisms, we used a global proteomic determination of liver protein profiles in laboratory adult female mice whose litter size had been either reduced or enlarged after birth. This litter size manipulation was expected to affect females by either raising or decreasing their current reproductive effort. At the same time, global parameters and levels of oxidative stress were also measured in all females.

**Results:**

Based on plasma analyses, females with enlarged litters exhibited increased levels of oxidative stress at the date of weaning compared to females with reduced litters, while no significant difference was found between both the latter groups and control females. None of the liver proteins related to oxidative balance were significantly affected by the experimental treatment. In contrast, the liver protein profiles of females with enlarged and reduced litters suggested that calcium metabolism and cell growth regulation were negatively affected by changes in the number of pup reared.

**Conclusions:**

Plasma oxidative stress levels in reproductive mice revealed that the degree of investment in reproduction can actually incur a cost in terms of plasmatic oxidative stress, their initial investment in reproduction being close to maximum and remaining at a same level when the energy demand of lactation is reduced. Liver proteomic profiles in reproductive females show that hepatic oxidative stress is unlikely to be involved in the cost of reproduction. Reproductive females rather exhibited liver protein profiles similar to those previously described in laboratory ageing mice, thus suggesting that hepatic cell pro-ageing processes may be involved in the cost of reproduction. Overall, our data illustrate how a proteomic approach can unravel new mechanisms sustaining life-history trade-offs, and reproduction costs in particular.

## Introduction

Life history theory has been said to explain the incredibly wide range of biological designs that allow organisms to successfully reproduce [1,2]. The main basis of this theory is that the limited resource availability entails an obligation to share it between different life history traits (the latter are often simplistically restricted to growth, reproduction and lifespan). Within this framework, the so-called cost of reproduction has attracted most of our attention in the past [3-5] because the individuals that reproduce the most are also the most likely to favour evolutionary changes through intergenerational genes transfer. For some time now, the cost of current reproduction has been described as an ultimate consequence, by either a reduction of future reproductive success [6] or a decreased survival rate of reproductive adults [2,7]. The fact that humans do not escape this reproduction *vs.* lifespan trade-off [8,9] underlines its widespread importance in evolution. However, when studies started to question the nature of the mechanisms sustaining reproductive cost, it rapidly became clear that the theory of a simple resource allocation trade-off (i.e. the competitive allocation of limited energy between body maintenance and longevity assurance on the one hand, and reproductive processes on the other [10,11]) was often unsatisfactory [12-14]. The non-energy based, proximate explanations of reproductive cost that then emerged largely improved the initial Y-shape trade-off models by highlighting the pleiotropic effects of hormones, the importance of cell signalling pathways and genetics, or the adverse impact of reproduction on body maintenance through oxidative stress [3,4,13,15-18]. However, the extent to which oxidative stress affects reproductive cost remains under debate [5,19]. For example, the assumption that oxidative stress increases in the liver of reproductive female mice has recently been challenged [20,21]. Because trade-offs may occur at functional levels ranging from cellular, tissue, physiological, and individual (physiology) to population/evolutionary (genetic), their detection remains a key challenge in evolutionary studies and is crucial to better explain why physiological and evolutionary trade-offs do not always match [22,23].

Proteins are main actors of physiological processes such as metabolism, development and homeostasis, so their screening via proteomics methodologies can be seen as a recent technical revolution for biological and medical research [24]. By unravelling the expression of the proteome, both qualitatively (protein identification and characterization of post-translational modifications) and quantitatively (expression level of each protein), proteomics notably make it possible to establish a link between the genome and the phenotype of an organism [25-27]. The proteome not only reflects the genomic information inherited from the parents and shaped by evolutionary processes, but is also influenced by environmental and developmental conditions, incorporating for example non-genetic inheritance modulation [28]. As such, the proteome should directly reflect individual phenotypes [29], the main level at which selection occurs [30]. In addition, the more recent progress in instrumentation and the increase in bioinformatic resources permit the analysis of proteins from almost any species, even non-sequenced [31], and have thus increased attractiveness of proteomics in several evolutionary biology fields [25] such as population dynamics [32], speciation [33], phylogenetics [29], or phenotypic plasticity [34,35].

To our knowledge, none of the aforementioned studies used proteomics to explore the nature of the mechanisms sustaining evolutionary trade-offs. We therefore aimed to do so in reproducing female mice that had previously been subjected to an experimental litter size manipulation. Litter size manipulation has indeed been proved to be an efficient method to assess the cost of reproduction in small mammals [36]. From previous studies, the hepatic metabolic functions of reproducing mice are expected to be stimulated because of the need to process an increased quantity of absorbed nutrients [37,38]. This would imply an enhanced function of the mitochondrial respiratory chain thus generating ROS and causing possibly an increase in hepatic oxidative stress. However, recent data suggest that oxidative stress does not increase with enhanced reproductive effort during lactation [20]. Therefore, we analyzed here the liver proteome of lactating mice not only to gather complementary data regarding the expression levels of antioxidant and metabolic liver proteins, but also and above all to uncover previously unknown changes that could represent/reflect new mechanisms (e.g. involving either metabolic or cell-related pathways) that could underlie the reproduction trade-off.

## Results

### Effect of litter size manipulation on mother and offspring

The impact of litter size manipulation on reproduction was evaluated on both offspring and mothers (Table 1). Adult female body mass did not differ among groups, either before litter size manipulation or at the end of the experiment. Table 1 also shows that there were no significant differences in litter size and litter mass among the three experimental groups before litter size manipulation (mean litter size across all groups: 7.4 ± 0.4 pups; mean litter mass, 9.9 ± 0.7 g, F_2,11_ = 0.36, P = 0.97). Our experiment successfully created three groups of females weaning significantly different numbers of offspring (Table 1). Post hoc tests showed that females with enlarged litters (FEL) were weaning significantly larger litter sizes than females with reduced litters (FRL) (Bonferroni post hoc, P = 0.004). The size of litters for Control females was midway between the sizes of the two other groups, and was not significantly different from them (Bonferroni post hoc, Control *vs.* FRL: P = 0.122 and Control *vs.* FEL: P = 0.181). At the end of the experiment, offspring exhibited a body mass which was not significantly different in relation to experimental litter size (Table 1), despite a trend towards heavier pups in Reduced litters.

**Table 1:** ANOVAs for Global parameters in litters and reproductive adult females

**Variable**	**Covariates**	**Repeated factor**	**Random factor**	**Control litters**	**Enlarged litters**	**Reduced litters**		**P-value**
	**P-value**	**P-value**	**P-value**	**(n = 4)**	**(n = 4)**	**(n = 4)**		
	**[Estimates]**	**[Estimates]**	**[Estimates]**	**[Estimates]**	**[Estimates]**	**[Estimates]**		
**(a) Litter parameters**								
**Initial litter size (pup number)**				8.3 ± 0.8	6.8 ± 0.5	7.3 ± 0.9	F_2,11_ = 1.61	0.253
				[0]	[-2.0 ± 1.2]	[-0.5 ± 1.2]		
**Final litter size (pup number)**				7.0 ± 0.9	9.3 ± 0.6	4.5 ± 0.7	F_2,11_ = 5.97	0.022
				[0]	[1.0 ± 1.1]	[-2.8 ± 1.1]		
**Final Litter mass (g)**	Final litter size			49.9 ± 4.0	45.4 ± 4.7	59.0 ± 5.3	F_2,11_ = 1.35	0.313
	P = 0.001			[0]	[-4.5 ± 5.8]	[9.1 ± 7.2]		
	[8.3 ± 1.6]							
**(b) Adult female parameters**								
**Initial body mass (g)**	Initial litter mass	Stage	Individual	27.4 ± 2.6	28.5 ± 2.6	31.7 ± 2.6	F_2,8.2_ = 4.00	0.061
				26.5 ± 2.6	27.7 ± 2.6	30.8 ± 2.6		
**Final body mass (g)**	P = 0.040	P = 0.225		27.0 ± 2.5	28.1 ± 2.6	31.2 ± 2.5		
**Mean female body mass (g)**	[0.2 ± 0.1]	[3.5 ± 2.8]	[4.1 ± 3.6]	[0]	[1.40 ± 1.8]	[4.9 ± 1.8]		
**Final oxidative stress (mg H2O2/mL)**	Antioxidant capacity			10.5 ± 0.7	12.4 ± 0.7	10.0 ± 0.7	F_2,11_ = 5.57	0,030
	P = 0.002			[0]	[1.9 ± 0.8]	[-0.8 ± 0.8]		
	[0.1 ± 0.1]							

Results of ANOVAs for global parameters in litters (a) and reproductive adult females (b) subjected to litter size manipulation (Control, FEL and FRL groups). “Initial” refers to pre-reproduction (i.e. 3 weeks before pairing) while “Final” refers to offspring emancipation (Day 21) characteristics. Analysis of variation in adult female body mass over time and treatment was done using a Mixed Model (see Statistic description for details).

### Oxidative analysis of maternal plasma samples

**Figure 1:**
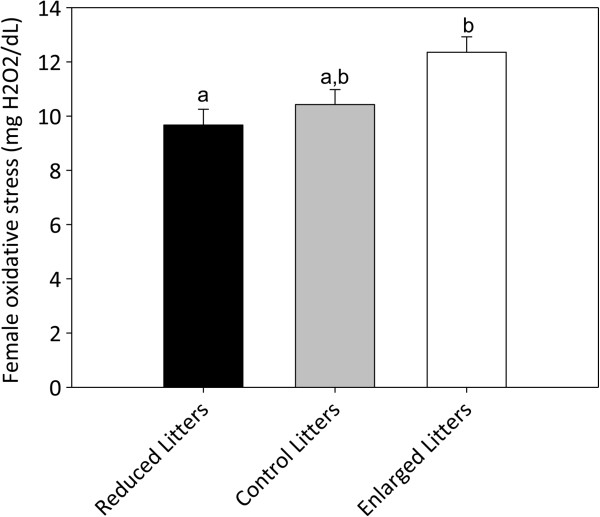
**Plasma oxidative stress (mean ± SE) in reproductive female mice.** Females were sampled at the end of the reproduction event. Litter sizes were either experimentally reduced, enlarged or unchanged (Control). Each group has a sample size of 4 individuals. Oxidative damage levels differed significantly (ANOVA) between females with enlarged and reduced litters (indicated by different letters). See text for detailed statistical analysis.

Plasma oxidative stress levels of reproductive females (dROM plasma levels controlled for OXY antioxidant plasmatic capacity) were measured when the offspring were separated from the mother (day 21). Levels of oxidative stress differed only between females from the FEL and FRL groups (Bonferroni post hoc, P = 0.037, other tests P > 0.123; Table 1, Figure 1).

### Proteomic analysis of maternal liver samples

ANOVAs conducted separately on the relative intensity of each of the 287 protein spots revealed that only 7 were found to be significantly affected by the litter size manipulation (all P < 0.05, Table 2). The 9 proteins identified in these 7 differential gel spots included indolethanolamine N-methyltransferase, fructose-1,6-biphosphatase 1, Glycine N-methyltransferase and malate dehydrogenase 1, alpha-enolase and adenosylhomocysteinase, dimethylarginine dimethylaminohydrolase 1, selenium-binding protein 1, and regucalcin (for details regarding mass spectrometry-based identifications of the proteins contained in all gel spots, see Additional files 1 and 2: Figure S1 and Table S1). It is important to note that several hepatic proteins directly concerned with the control of the oxidative balance were identified in some gel spots (see Table 2 and Additional file 2: Table S1), but none of them were found to be affected by litter size manipulation (see Table 2 for separate ANOVA P values).

**Table 2:** Results of separate ANOVAs conducted on each protein spot detected.

**Spot N°**	**Dependent variables**	**Acc. N°**	**Biological activity**	**F**	***P (ANOVA)***
657	Indolethylamine N-methyltransferase	gi|731019	Methylation, ageing	7.80	**0.011**
516	Fructose-1,6-biphosphatase 1	gi|14547989	Carbohydrate metabolism, calcium chelation	7.58	**0.012**
570	Glycine N-methytransferase	gi|15679953	Biogenesis, methylation, carbohydrate metabolism	6.12	**0.021**
	Malate dehydrogenase 1	gi|148675904			
422	Alpha-enolase	gi|13637776	Cell growth, biogenesis, methylation	5.19	**0.032**
	Adenosylhomocysteinase	gi|21431841			
553	Dimethylarginine dimethylaminohydrolase 1	gi|45476974	Regulation of nitric oxide generation	4.83	**0.038**
383	Selenium-binding protein 1	gi|148840436	Cell differentiation	4.51	**0.044**
748	Regucalcin	gi|2498920	Calcium homeostasis, ageing	4.46	**0.045**
355/365	Catalase	gi|157951741	Response to oxidative stress	0.01/0.03	0.99/0.97
437	Ndufs 2 protein	gi|13278096	Response to oxidative stress	0.08	0.93
637	Hydroxyacyl glutathione hydrolase	gi|13435786	Response to oxidative stress	1.10	0.37
	Carbonic anhydrase 3	gi|148673185			
663/672	Peroxiredoxin 6	gi|6671549	Response to oxidative stress	0.73/0.77	0.51/0.49
702/791	Peroxiredoxin 1	gi|547923	Response to oxidative stress	0.73/0.38	0.51/0.69
843/850	Glutathione transferase	gi|193703	Response to oxidative stress	0.05/0.11	0.95/0.90
681/683	Glutathione transferase zeta 1	gi|148670978	Response to oxidative stress	0.91/0.06	0.44/0.94
702	SOD 2 protein	gi|17390379	Response to oxidative stress	0.73	0.51
665/853	Glutathione S transferase Mu 1	gi|121716	Response to oxidative stress	0.17/0.42	0.85/0.67
665/673	Glutathione S transferase Mu 2	gi|121718	Response to oxidative stress	0.17/0.80	0.85/0.48
853	Glutathione S transferase Mu 3	gi|121720	Response to oxidative stress	0.42	0.67
850	Glutathione S transferase Kappa 1	gi|47116757	Response to oxidative stress	0.11	0.90
680	Glutathione S transferase theta-1	gi|160298219	Response to oxidative stress	0.68	0.53
791	Glutathione S-transferase P 1	gi|121747	Response to oxidative stress	0.38	0.69
681	Thioredoxin-dependent peroxide reductase	gi|126986	Response to oxidative stress	0.91	0.44
639	Carbonic anhydrase 2	gi|146345383	Response to oxidative stress	0.80	0.48
386/390/393/633/637/647/649/778	Carbonic anhydrase 3	gi|148673185	Response to oxidative stress	0.22/0.07/0.19/0.29/1.10/0.10/0.58/0.36	0.81/0.93/0.83/0.76/0.37/0.90/0.58/0.71

Data were collected in reproductive females belonging to FEL, FRL and Control groups (n = 4 per group). Multiple ANOVAs were used to determine the spots that were likely to be affected by the experimental treatment, and that then entered the subsequent statistical analysis (see Materials and methods for details). Only 7 protein spots containing 9 different proteins were found to be significantly affected by the litter manipulation (in bold). The proteins related to oxidative balance that were detected in the proteomic analysis of the liver samples but which were apparently not affected by litter size are also indicated. Spot N° correspond to the protein spots reported in Additional file 1: Figure S1. The biological activity of proteins as determined using the so-called biological process ontologies (http://www.geneontology.org/) were automatically extracted using the MSDA software suite (https://msda.unistra.fr), and complemented with literature examination.

**Figure 2:**
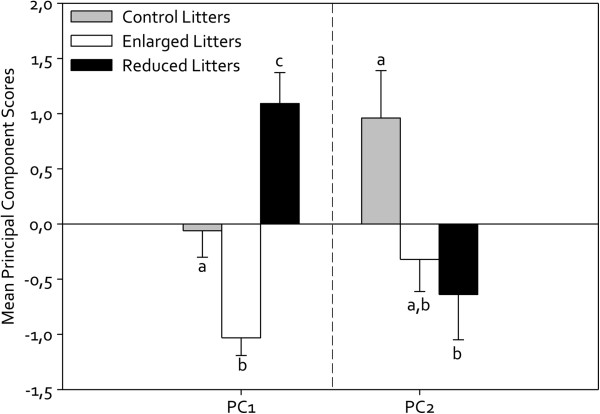
**Principal Component Analysis of liver proteomics data.** Modulation by litter size manipulation of female liver protein expression related to calcium metabolism and ageing (PC1) and to cell growth regulation and biogenesis (PC2). Principal Component Analysis was conducted on 7 protein spots (i.e. 9 proteins). See text for statistical details. Bars (±SE) labelled with different letters are significantly different.

A PCA analysis was run using the 7 differential protein spots (i.e. the 9 differential proteins), and two components (PC1 and PC2) with eigenvalues over 1 were obtained, explaining 77% of the total variance after rotation. Table 3 indicates the loading values of each protein. Given our relatively small sample size, only protein loadings over 0.6 were taken into account (see [39]), and our results were thus reduced to 6 protein spots and 8 proteins. Taking into account the main biological activity (based on biological process GO ontologies and literature examination; see Table 2 for details) of the proteins that cluster on the same component, our results suggest that PC1 was related to calcium metabolism and ageing markers while PC2 represented cell growth and biogenesis regulation. Glucose metabolism-related proteins were also present in both components. PC1 and PC2 indices were further used in an ANOVA analysis to determine how the two components were affected by litter size manipulation (Table 4). We found a significant effect of litter size manipulation on PC1 (calcium metabolism and ageing), with FEL having the lowest score, FRL having the highest and Control females being intermediate (Figure 2, Bonferroni post-hoc, all P ≤ 0.05). There was no significant effect of Final litter size or Final female body mass on the output (Table 4). PC2 component was also found to respond significantly to litter size manipulation, but only FRL differed significantly from Control individuals (Bonferroni post-hoc, P = 0.048, Figure 2) while other group comparisons were non-significant (Bonferroni post-hoc, all P > 0.13; Table 4).

**Table 3:** Principal Component Analysis conducted on liver protein expression

**Variables**	**PC1**	**PC2**
	**Calcium metabolism and ageing**	**Cell growth and biogenesis**
Regucalcin (spot N°748)	0.86	
Fructose-1,6-biphosphatase 1 (spot N°516)	0.82	
Indolethylamine N-methyltransferase (spot N°657)	0.74	
Glycine N-methytransferase and/or Malate dehydrogenase 1 (spot N°570)		0.93
Alpha-enolase and/or Adenosylhomocysteinase (spot N°422)		0.89
Selenium-binding protein 1 (spot N°383)		-0.72
**Eigenvalues**	**2.77**	**1.86**
**% of variance**	**46.2**	**31.1**

Principal Component Analysis was run from liver protein expression data of 12 reproductive adult females after litter size manipulation. Spot N° correspond to those reported in Additional file 1: Figure S1.

**Table 4:** Separate ANOVAs conducted on PC1 and PC2 values of individual reproductive female mice

	**PC1**			**PC2**		
	**Calcium metabolism and ageing**			**Cell growth regulation**		
	**F**	**d.f.**	**P**	**F**	**d.f.**	**P**
**Fixed effects**						
Litter size manipulation	20.68	2, 11	**<0.001**	4.89	2, 11	**0.036**
**Covariates**						
Final litter size	0.20	1, 11	0,67	1.81	1, 11	0.22
Final female body mass	0.01	1, 11	0.91	1,15	1, 11	0,32

Final litter size and female body mass at the end of the experiment (n = 12, 4 females in each group) were initially included as covariates (P values indicated in brackets) but were dropped sequentially to obtain the final model. Bold values indicate significant effects.

**Figure 3:**
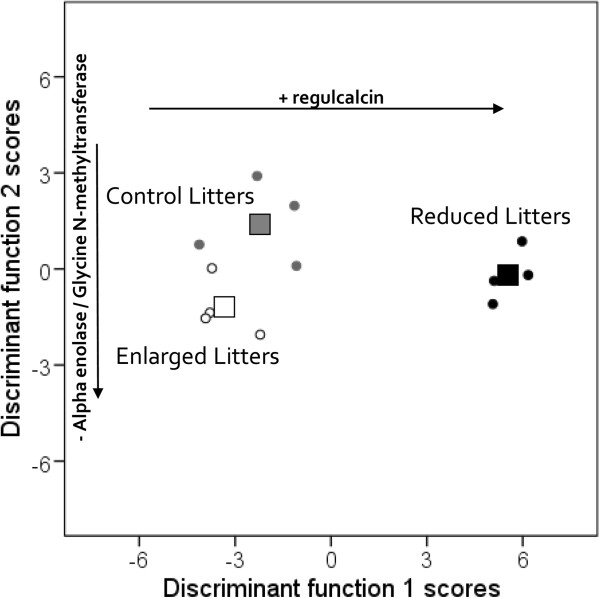
**Combined-group plot of canonical discriminant function scores.** Both individual discriminant scores (circles) and group centroids (squares) are shown. The x-axis shows that function 1 best discriminates the females with reduced litters on one side, and the two other groups on the other side, while the y-axis shows that function 2 separates the Control from the two other groups, but for a lower difference than function 1. Function 1 is mainly driven by an increased expression of regucalcin in females with reduced litters (horizontal arrow), while function 2 is defined by a reduced expression of alpha-enolase and glycine N-methyltransferase/malate dehydrogenase in females with enlarged litters (horizontal arrow).

In addition to the PCA analysis, we followed up with a Discriminant Analysis that was restricted to the 6 protein spots (containing 8 differential proteins) that were found to be affected by the treatment during the PCA-ANOVA analysis. It reveals two discriminant functions: function 1 explained 98.7% of the variance, whilst function 2 accounted for only 1.3%. When combined, these two functions significantly discriminate between the three experimental groups (Wilk’s lambda, = 0.017, Chi-Square = 26.35, *P* = 0.010), but removing the first function indicated that the second alone did not (Wilk’s lambda, = 0.383, Chi-Square = 6.23, *P* = 0.28). Standardized Canonical Discriminant Function Coefficients indicated how the dependent variables contribute to each function: regucalcin (spot N°748) loaded more for function 1 (r = 0.21), while alpha-enolase/adenosylhomocysteinase (spot N°422; r = 0.82) and glycine N-methyltransferase/malate dehydrogenase (spot N°570; r = 0.59) loaded more for function 2 (indolethylamine spot N°657, r = 0.35; fructose-1,6 biphosphatase spot N° 516, r = 0.33; selenium binding protein spot N°383, r = -0.32). The FRL group is differentiated from the two other experimental groups by function 1, while function 2 discriminates between the Control and FEL groups, with the FRL group positioned between them (Figure 3).

Merging PCA-ANOVA and Discriminant analyses indicates that the females who raised a reduced litter had higher quantities of regucalcin than the two other groups, while the females from the FEL group exhibited lower levels of alpha-enolase/adenosylhomocysteinase and glycine N-methyltransferase/malate dehydrogenase than Control females.

## Discussion

Life-history theory predicts that processes such as growth or reproduction will limit individual lifespan [1,40] by reducing the amount of energy that can be devoted to organism maintenance [11]. However, other non-energy based mechanisms may underlie the cost of reproduction [22,41]. Our study lends credibility to this idea, as the proteomic patterns we obtained suggest that oxidative stress is not necessarily observed at the tissue level despite its presence at the plasma level. The sole determination of systemic oxidative stress may thus be insufficient, and may blur our perception of reproductive costs. In fact, the use of proteomics enabled us to highlight both non-energy and non-oxidative stress mechanisms as new pathways through which reproductive costs may be expressed.

### Cost of reproduction and oxidative stress

Oxidative stress reflects an imbalance between reactive oxygen species (ROS) production (mainly attributable to mitochondria during energy processing) and antioxidant defences/repairing processes that have the ability to either buffer ROS or counterbalance their negative effects on bio-molecules [42]. In mice, a plethora of studies have been conducted to experimentally test the oxidative mediation of reproductive cost (e.g. [3,4,21,36] and references therein). There is a debate about reproductive costs, and whether they are attributable to reproduction alone or rather to the effort required for raising more young [3,19,20]. By directly testing the impact of the degree of investment in reproduction, our litter size manipulation underlined that rearing a higher number of pups than initially programmed triggers significantly higher levels of plasmatic oxidative stress than rearing a lower number of pups than initially programmed. Thus, the degree of investment in reproduction can actually incur a cost in terms of plasmatic oxidative stress. Therefore, reproduction is more likely to affect body maintenance when females with enlarged litters (FEL) are no longer able to tailor their initial level of investment to their experimentally enlarged litter size. Under natural conditions, reproduction may then become particularly costly in terms of future adult fitness only when females have to face unexpected and more demanding reproductive conditions [43], either due to external (i.e. sudden environmental change [21,44,45]) or internal factors (i.e. change in female conditions such as energy status [44,46]). Still, oxidative stress in this study only differed significantly between females with reduced litters (FRL) and FEL (i.e. levels of Control females were intermediate and were not significantly different). This supports the idea that initial investment in reproduction is close to the maximal female reproductive potential (as FEL do not suffer more oxidative stress than Control ones), and remains close to the level initially programmed (as FRL do not suffer less oxidative stress than Control ones). This also lends support to a previous study, which showed that female bank voles raising a reduced number of offspring had the same survival rate or fecundity as Control females [43]. It is interesting to note that body mass did not seem to be a reliable marker of reproductive investment in mice, since females gained mass throughout reproduction in all groups (see also [47]).

The characterization of systemic oxidative stress was an important step in our understanding of non-obligatory energy-derived trade-offs [15,48]. Our proteomic strategy provided a broader picture of the oxidative state at the tissue level, which may be of key importance before drawing a final conclusion on the role of oxidative stress in any trade-offs [49]. However, a great number of proteins involved in the liver oxidative balance regulation (e.g. superoxide dismutase or other antioxidant proteins) were analysed here using proteomics, but we did not detect any significant change in their levels following litter size manipulation (see Table 2 and Additional file 2: Table S1). Given the small sample size, our results have to be carefully interpreted but they suggest that hepatic oxidative stress is in our case unlikely to be involved in the cost of reproduction. This hypothesis is in accordance with recent studies in which no increase was observed in oxidative stress in the liver of wild mice after litter size manipulation, despite the use of multiple proxies to measure oxidative balance [20,21]. In the aforementioned studies, superoxide dismutase activity had even increased and protein carbonylation and oxidation levels had decreased in the liver, indicating a rather low level of oxidative stress in females raising an enlarged litter size. In our study, the lack of a significant effect on such antioxidant proteins in the liver is unlikely to be attributable to a difference in experimental designs, since Garratt et al. used a comparable litter size manipulation, with similar average litter sizes (i.e. around 6 pups). Nevertheless, liver oxidative stress cannot be definitively ruled out as a reproductive cost by our data. For instance, antioxidant parameters like glutathione peroxidase and peroxiredoxins have previously been shown to vary with age in mice using a proteomic approach [50]. It may therefore be possible that a deleterious effect of our treatment would only occur in old reproductive females. In addition, the timing of sampling may be of importance in the detection of oxidative stress as a cost of reproduction related to litter size. Previous measurement of protein oxidation in mice indicated that levels of oxidative damage increased with litter size at peak lactation (day 17 after parturition) but not at weaning (day 28) [21]. Finally, an alternative explanation remains, namely that enzymatic activity and protein expression are not directly linked. Therefore, while proteomics may highlight new protein targets, definitive conclusions about the implication of a given enzymatic pathway cannot be drawn without carrying out studies that perform functional tests or measure the proportion of intact/damaged protein forms (i.e. carbonylation levels of enzymes, [51]).

### Non oxidative-related proteins affected by litter size manipulation

As regards litter size manipulation, our proteomic approach highlighted new mechanisms (i.e. other than oxidative stress) that may be involved in the cost of reproduction. Interestingly, some of these proteins are related to ageing. One of our most striking findings was that the levels of regucalcin (or senescence marker protein-30) were lower in the FEL than FRL group. A comparable reduction of regucalcin levels was previously observed in a senescence-accelerated mice strain and also in ageing rats [50,52]. Regucalcin plays a central role in Ca^2+^-signalling pathways (e.g. cell apoptosis) and Ca^2+^-dependent regulation of protein activities and is involved in the protection of cells and organs from various deleterious factors, including oxidative stress [53]. Finally, knockout mice lacking regucalcin have a shortened lifespan [54], suggesting that this protein plays a corner-stone role in organism ageing and survival. In addition to being repressed (expression levels) in the FEL group of this study, regucalcin actually meets all the biological requirements for a component of one of the mechanisms sustaining the ageing cost of reproduction: (i) regucalcin is partly localized in the mitochondria (a central actor in ageing processes), (ii) its protein sequence appears to be highly conserved among vertebrates, and (iii) its expression is modulated by a myriad of different factors from hormones to lipopolysaccharide, all potential mediators of life-history traits. Thus, one can hypothesize a putative broader evolutionary role of regucalcin in any life-history trade-off implying ageing. This hypothesis deserves further studies in mammals but also in other taxa.

Our proteomic results also underline the modulated expression of a few additional proteins. The levels of glycine N-methyltransferase/malate dehydrogenase were down-regulated in females of the FEL compared to the Control group (Discriminant Analysis). This type of decrease in glycine N-methyltransferase and malate dehydrogenase was previously found either in senescence-accelerated mice strains [50] or simply with age ([55]; see also [56] for a deleterious ageing impact on adenosylhomocysteinase levels), reinforcing the potential pro-ageing effect of our experimental litter enlargement. Decreased activities of mitochondrial proteins involved in oxidative phosphorylation or in the transfer of co-enzymes through the mitochondrial membrane are actually concomitant to ageing [57], suggesting a lower efficiency in the mitochondrial conversion of energy in ATP. However, malate dehydrogenase is an enzyme involved in the tricarboxylic acid cycle producing NADH, an essential cofactor for mitochondrial oxidative phosphorylations. Malate dehydrogenase is also involved in fatty acid synthesis. Additionally, glycine N-methyltransferase plays a role in amino acid metabolism and controls methylation processes. It is therefore possible that the reduced levels of glycine N-methyltransferase/malate dehydrogenase in females from both the FRL and FEL groups may also be explained by other less or non-costly mechanisms such as a simple metabolic switch between the use of lipid fuels and amino acids.

Selenium-binding protein acts as a tumor-suppressor and is expressed more in healthy tissues than in cancer cells [58], thereby forming a possible indicator for the long-term effect of reproductive investment on adult survival. When compared to Control females, selenium-binding protein was found to be up-regulated in females for which litter size was reduced (PCA analysis). This suggests that raising less young than initially programmed would allow the mother to set up a better body protection. Interestingly, in addition to malate dehydrogenase, our analysis indicated that the expression of two other proteins involved in carbohydrate metabolism were altered by litter size manipulations, with either a decreased level in the FEL group (alpha-enolase) or an increased level in the FRL group (fructose-1,6-biphosphatase 1), thus indicating a possible constraining effect of reproduction effort on the metabolism of carbohydrate fuels, or at least on these proteins. This indicates a potential alteration of glucose homeostasis following the mismatch between the female’s projected reproductive investment and the actual size of the litter she has to rear. A similar observation has been made in 50-week old control and senescence-accelerated mice strains [50]. Importantly, as a putative ecological consequence of these changes in protein expression, fructose-1,6-biphosphatase 1 regulates blood glucose and a defect in its activity may ultimately lead to altered brain functions such as memory [59], a function of key importance in wild animals both in terms of foraging efficiency and predator avoidance [60]. Overall, it points out that altered glucose metabolism may also be one of the pathways through which reproduction is traded-off against ageing and the future survival/fecundity prospects of adults. We therefore encourage future studies to include measurements of glucose metabolism (and other energy metabolic pathways) as an interesting (and easily measured) variable to evaluate the consequences of reproduction. This is in line with the recent outlining of a potential connection between reproduction, fat metabolism and longevity [61].

## Conclusions

In summary, our results show that enlarging or reducing litter sizes in reproductive mice does not trigger any significant change in systemic oxidative stress levels in comparison to a control reproductive state. This suggests that mice initial investment in reproduction is close to a maximum and remains at a same level even if the energy demand of lactation is reduced. However, the significant difference between females with a reduced and enlarged litter size reveals that the degree of investment in reproduction can actually incur a cost in terms of plasmatic oxidative stress. Our proteomic data further show that hepatic oxidative stress is unlikely to be involved in the cost of reproduction, as expression levels of a series of proteins involved in the liver oxidative stress response are not affected by litter size manipulation. Importantly, liver proteomic data also enabled us to uncover that pro-ageing processes are new mechanisms underlying the cost of reproduction. As a consequence, this should generate interest of evolutionary biologists and ecophysiologists in the use of a proteomic approach to complement their studies of the mechanisms sustaining life-history trade-offs. The collection of data for proteomes and their variation among individuals responding to environmental challenges or stressful situations may reveal particular cellular or physiological networks which have a closer link with the individual phenotype. This multidisciplinary field of investigation opens up real possibilities for a more integrated view of evolutionary processes.

## Materials and methods

### Experimental procedures

The experiment was conducted on females C57 black 6 (C57BL/6 J) from our own laboratory population. The study complied with the ‘Principles of Animal Care’ publication no.86-23, revised 1985 of the National Institute of Health, and with current legislation (L87-848) on animal experimentation in France. Animals were kept under constant environmental conditions (26 ± 1°C, 12 L: 12 D light cycle) and food (SAFE A03) and water were provided *ad libitum*. The litter size manipulation experiment was conducted on 12 primiparous females aged 5 months, which were mated with adult males of the same strain (6–9 months old). We created 3 experimental groups of 4 females as follows: (i) Control females with unmodified litter size, (ii) females with enlarged litters (FEL) and (iii) females with reduced litters (FRL). In the two latter groups, litter size was increased or reduced by two offspring, respectively, two days after parturition.

### Body mass and physiological measurements

The body mass of both adult females and the entire litter were measured every morning (±0.1 g) from birth (day 1) to offspring emancipation (day 21, i.e. when the pups were put in a different cage). At that date, the reproductive females were culled by cervical dislocation (in the morning, between 9 and 11 a.m.) and their livers (3 aliquots of 300 mg) were snap frozen in liquid nitrogen using Nalgene™ cryotubes and stored at -80°C until proteomic analysis. Just before pair formation (i.e. 3 weeks before parturition), adult females were blood sampled (60 μL) as indicated in Stier et al. [4] to obtain initial values of the oxidative status. A second blood sample (final oxidative status) was taken just before culling the reproductive females (at day 21 after parturition). That date corresponds to a period of high female energy demand [36]. Plasma was collected after centrifugation (3000 g, 10 min at 4°C) and stored at -80°C.

The oxidative status of the reproductive females was assessed from the plasma using the OXY-adsorbent and d-ROMs tests (Diacron International s.r.l., Italy). These measurements have previously been performed on frozen mouse plasma [4] and are presented in detail in Costantini et al. [62]. Briefly, the OXY-absorbent test quantifies the antioxidant buffering ability of the whole plasma against the oxidative activity of hypochlorous acid, providing a picture of mainly the non-enzymatic antioxidant capacity of the plasma. The d-ROM test evaluates the plasmatic levels of early lipid and protein oxidative damages. The OXY values are expressed as mM of neutralized HclO, while the d-ROM values are measured in mg of H2O2 equivalent. Plasma samples were run in duplicate in a single OXY or d-ROM run, and intra-individual variation for both measures was low (2.1 ± 0.4% and 2.9 ± 0.9%, respectively).

### Proteomic methodology: 2D-DIGE/MS experiment

Unless otherwise specified, all chemicals and reagents were purchased from Sigma Aldrich (St. Louis, MO, USA).

#### Protein extraction

Frozen liver samples from the 3 experimental groups were ground in liquid nitrogen using a laboratory ball mill (Mikrodismembrator, Sartorius). ~10 mg of the resulting powders were solubilized in 400 μL of 8 M Urea, 2 M Thiourea, 4% Chaps, 1% dithiothreitol, Triton X100 0.5%, TLCK 0.05% and 0.02 to 2 mM protease inhibitors then sonicated on ice (10 s, 135 watts). Proteins were acetone-precipitated overnight at -20°C using 9 volumes of cold acetone. After centrifugation (14 min, 4°C, 14000 g), supernatants were discarded and protein pellets were vacuum-dried (Speedvac, Thermoscientific) and dissolved in a 7 M Urea, 2 M Thiourea, 30 mM Tris (pH 8.5) and 4% Chaps buffer. The pH was then adjusted to 8.5, and homogeneization was completed by sonication on ice (10 s, 135 watts). Protein concentrations were determined using the Bio-Rad Protein Assay (BioRad, Hercules, CA, USA). Protein profile checking was achieved for each sample after separation on a 12% SDS-PAGE acrylamide gel (20 μg loaded; 50 V for 30 min and then 100 V to complete migration) and Coomassie blue staining. Similarity of protein profiles between all samples was then verified prior to quantitative DIGE analyses.

#### Protein labelling

Protein samples were labelled using a CyDye DIGE Fluor Minimal Dye Labeling Kit (GE HealthCare, Uppsala, Sweden). After the reconstitution of CyDyes in anhydrous N,N-dimethylformamide, 400 pmol of Cy3 and Cy5 were used to randomly label 50 μg of protein samples from the different groups, and 2.4 nmol of Cy2 were used to label a mixture of all the samples (25 μg each) that was used as an internal standard. After incubation in the dark for 30 min on ice, protein labelling was quenched by addition of 10 mM lysine and incubation in the dark for 10 min on ice.

#### 2D gel electrophoresis

Prior to 2D gel electrophoresis, the multiplexing of samples from the Control (non-manipulated), FRL, and FEL group was randomized to avoid any bias. Briefly, 50 μg of Cy2, Cy3 and Cy5-labelled protein samples were mixed and diluted with 7 M urea, 2 M thiourea, 2% Chaps, 2% DTT, 2% ampholytes (Amersham Pharmacia-Biotech, Uppsala, Sweden), and a trace of bromophenol blue to a total volume of 400 μL. Proteins were then loaded onto 18 cm pH3-10 non-linear immobilized pH gradient strips (IPG Ready strip, Biorad, Hercules, CA, USA), and left in the dark for passive rehydration over 2 h 30. Active rehydration was then performed overnight at 50 V using a Protean IEF cell (Biorad, Hercules, CA, USA), and subsequent isoelectric focusing (IEF) was performed using voltage gradient steps (from 0 to 200 V in 1 h, from 200 to 1000 V in 4 h, from 1000 to 5000 V in 16 h, then 5000 V for 7 h) with a total focusing time of 85000 Vh. Focused proteins were then reduced and alkylated by equilibration of IPG strips (30 min in 1% DTT, 6 M Urea, 50 mM Tris pH 8.8, 30% glycerol and 2% SDS followed by 30 min in 2.5% iodoacetamide, 6 M Urea, 50 mM Tris pH 8.8, 30% glycerol and 2% SDS). IPG strips were then sealed onto 10% polyacrylamide SDS-PAGE gels (20 × 20 cm) with 0.5% agarose, and electrophoresis was carried out using a Protean II xi Cell (Biorad Hercules, CA, USA), applying 5 mA per gel for 1 h followed by 8 mA per gel for 8 h.

Another 2D-gel was performed in parallel in order to improve the quality of mass spectrometry-based protein identifications. It was loaded with a higher amount of proteins, i.e. 840 μg of the non-labelled internal standard, and was stained with Coomassie blue after electrophoresis.

#### 2D gel image acquisition and analysis

Following 2D gel electrophoresis, gels were washed with water and gel images were acquired using an Ettan DIGE Imager (GE HealthCare, USA) at 100 μm resolution (Ettan DIGE Imager, Ge Healthcare Uppsala, Sweden). Gel images were analyzed using Progenesis Samespots (v4.5, Nonlinear dynamics, Newcastle, UK). After automatic control of image quality and automatic alignment of images, minor corrections were manually applied to obtain a more accurate image alignment. The next steps in the Samespots analysis included background subtraction, the normalization of Cy3 and Cy5 spot volumes to those of corresponding Cy2 spots, and a correction based on 1) the calculation of the global distribution of all Cy3/Cy2 and Cy5/Cy2 ratios and 2) the determination of a global scaling factor for all gels. This normalization procedure enabled us to eliminate any possible inter-gel variations and provided accurate quantitative data.

#### Mass spectrometry-based analyses

Differential protein spots (see Statistics) were excised using an automated gel cutter (PROTEINEER sp, Bruker Daltonics, Bremen, Germany) and destaining, in-gel reduction and in-gel alkylation of proteins were performed using a Massprep Station (Waters, MicroMass, Manchester, UK). Briefly, destaining was carried out with 3×10 min wash cycles in 50 μL of 25 mM NH_4_HCO_3_ and 50 μL of acetonitrile, followed by a dehydration step (50 μL acetonitrile, 60°C, 5 min). The reduction step was performed at 60°C for 30 min in 50 μL of 10 mM DTT, 25 mM NH_4_HCO_3_, and the alkylation step for 30 min in 55 mM iodoacetamide, 25 mM NH_4_HCO_3_. After a washing step in 50 μL of 25 mM NH_4_HCO_3_ and 50 μL of acetonitrile for 10 min, gel spots were dehydrated in 50 μL of acetonitrile for 15 min. Proteins were in-gel digested using trypsin (Promega, Madison, WI, USA). 10 μL of a 12,5 ng/L trypsin solution in 25 mM NH_4_HCO_3_ were then added to the gel spots before incubation for 5 h at 37°C. The resulting peptides were then extracted using 30 μL of a 60% acetonitrile solution containing 0.1% of formic acid. Acetonitrile was removed by vacuum drying using a speedvac.

Tryptic peptides were analyzed on a 1200 series nanoHPLC-Chip system (Agilent Technologies, Palo Alto, CA, USA) coupled to an HCT™ Plus ion trap (Bruker Daltonics, Bremen, Germany). The Chip system was composed of a 40 nL trapping column (ZORBAX 300SB-C18, 4 mm, with a 5 μm particle size), a separation column (ZORBAX 300SB-C18, 43 mm × 75 μm, 5 μm) and a sprayer. The solvent system consisted of 2% acetonitrile, 0.1% HCOOH in water (solvent A) and 2% water, 0.1% formic acid in acetonitrile (solvent B). 6 μL of samples were injected onto the trapping column at a flow rate of 3.75 μL/min with solvent B. Peptides were eluted at a flow rate of 300 nL/min, according to the following gradient: t = 0 min 8% B, t = 7 min 40% B, t = 8 min, 70% B, t = 10 min 70% B. The mass spectrometer was operated with automatic switching between MS and MS/MS modes. The following voltages were set up: -1800 V (inlet), +147.3 V (outlet) and a skimmer voltage of +40 V. For mass spectrometry data acquisition, the scan speed was set at 8100 m/z per sec in the MS mode and 26000 m/z per sec in the MS/MS mode. Mass range was set at 250–2000 m/z in the MS mode and 50–2800 m/z in the MS/MS mode. The 3 most intense ions (doubly charged) were selected for fragmentation, and exclusion was set at 1 min or 2 spectra. The system was fully controlled by ChemStation (Rev B.01.035R1) and EsquireControl (v5.3) software (Agilent technologies and Bruker Daltonics, respectively).

#### Mass spectrometry data analysis and protein identifications

For protein identifications, MS/MS data were analyzed using two different algorithms, i.e. the Mascot™ v2.3.02 (Matrix Science, London, UK) installed on a local server and the OMSSA (Open Mass Spectrometry Search Algorithm) program (v2.1.7, [63]). Spectra were searched against a target-decoy version of the *Mus musculus* protein database downloaded from NCBInr (May 2011, 288050 target + decoy entries), with a mass tolerance of 0.25 Da in MS and MS/MS modes, and allowing a maximum of one trypsin missed cleavage. Optional modifications were set as follows: carbamidomethylation of cysteine residues, oxidation of methionine residues, and acetylation of protein N-termini. Database generation, OMSSA searches and automatic extraction of protein functional annotations (gene ontologies) were performed using our home-made “Mass Spectrometry Data Analysis” software suite [64].

We followed the guidelines for proteomic data publication [65,66], and to avoid any eventuality of poor quality data being considered, we applied stringent filtering criteria based on probability-based scoring of the identified peptides to obtain high confidence identifications. A target-decoy strategy was used to determine the false discovery rate of identifications [67]. This was achieved using Scaffold software (v3.0.7, Proteome software Inc., Portland, OR, USA). Single peptide-based protein identifications were validated when MS/MS ion scores were higher than 55 (Mascot) and when –logE values were higher than 8 (OMSSA). Multiple peptide-based protein identifications were validated when at least 2 peptides were detected with a MS/MS ion score higher than 20 (Mascot) and/or when –logE values were higher than -2 (OMSSA). Common contaminants such as keratin and trypsin were not considered. Among the different proteins that were identified in a given spot, only the major (more abundant) ones were considered to be responsible for variations of spot intensities. The determination of major proteins was performed following a “peptide counting” strategy: the higher the number of peptides assigned to a given protein, the more abundant this protein is. More precisely, and while taking into account the fact that tryptic sites are followed or not by a Proline, the possible missed cleavages, the adequate size of peptides for their detection by mass spectrometry (i.e. peptides of 4 to 31 amino acids on the basis of our data), and also the results from two distinct search algorithms, we compared the theoretical number of detectable tryptic peptides to the experimental number we identified. The theoretical number of detectable tryptic peptides was similar for major (53 ± 2) and minor (53 ± 8) proteins in a given gel spot, and major proteins were identified with five times more peptides than minor ones (27 ± 2% vs. only 5 ± 1% of the possible tryptic peptides, respectively).

### Statistical analysis

Impact of litter size manipulation on reproductive female body mass was checked using a Mixed Model Analysis, with body mass as the dependent variable and Experimental Group (Control, FEL and FRL) as a fixed factor. Initial litter mass (measured before litter manipulation) was used as a covariate to control for initial investment in reproduction, and Time (before and after litter size manipulation) as repeated factor. Individual identity was added as a random factor to control for pseudo-replication. Effect of the experimental litter size manipulation on final oxidative stress of the females was checked using a one-way ANOVA, with antioxidant capacity used as a covariate to assess the oxidative stress level after controlling for any variation in individual antioxidant level.

In addition, we tested whether manipulating female current reproductive effort may have incurred a cost for the offspring in terms of lower body mass growth. To do so, final litter mass differences were tested using one-way ANOVA, with Experimental Group as a fixed factor and final litter size as a covariate to control for the number of offspring present in the litter. The significant effect of litter manipulation and litter size was also checked using a similar one-way ANOVA.

The proteomic data encompassed 287 different protein spots revealed on 2D-gels and corresponded to a total of 419 different proteins. Indeed, one protein spot can contain several different proteins. We chose to conduct two complementary statistical analyses and expected their convergent results to help us identify the main proteins affected by our experimental design. The first of these statistical procedures analysed data using multiple ANOVAs to detect spots that were significantly different among the three experimental groups (Control, FEL and FRL groups). By doing so, we were able to determine, among the large number of protein spots, those that were of interest in relation to our experimental treatment. Using these ANOVAs allowed us to restrict the numbers of variables to 7 protein spots. In a second step, we conducted a Principal Component Analysis on the 7 selected protein spots with orthogonal rotation (varimax, see [68]). The aim was to obtain two components (PC1 and PC2) that would largely explain the total variance. PC1 and PC2 indices were then used in an ANOVA analysis, with Litter Size Manipulation as a fixed factor, and litter size after manipulation (Final Litter Size) and female body mass at the end of the experiment (Final Female Body Mass) as covariates to control for absolute litter size effect (regardless of litter size manipulation) and for individual differences in female quality, respectively. Non-significant terms were dropped sequentially to obtain the final model we present.

To further disentangle the roles played by each protein found in PC1 and PC2 in the discrimination of the experimental groups, we used a Discriminant Analysis [39]. Combining the results from both tests enabled us to focus more particularly on just three proteins (alpha-enolase, Glycine N-methyltransferase and regucalcin; see Results section).

All analyses were conducted on SPSS 18.0. Variance homogeneity and the normal distribution of residuals were checked in each ANOVA test. Kaiser-Meyer-Olkin Measure of Sampling Adequacy (KMO value > 0.55) and Bartlett’s test of sphericity (testing whether correlations between variables were large enough for PCA, *P* < 0.05) were verified in the Principal Component Analysis [39]. The determinant of the correlation matrix in the Principal Component Analysis was always found to be: d > 0.026. Means are given ± standard deviation. Significance threshold is *P* < 0.05.

## Competing interest

The authors declare that they have no competing interests.

## Authors’ contributions

FC designed the study and collected the data. FB designed and managed proteomics analyses. MIP, AS and DT performed the proteomic and oxidative stress measurements. FC, FB and AvD wrote the paper. All authors have read and approved the final version of the manuscript.

## Authors’ information

François Criscuolo and Fabrice Bertile are shared seniorship of the paper.

## Supplementary Material

Additional file 1: Figure S1 Representative 2D-gel image of mouse liver proteins. Significantly different protein spots according to ANOVA analysis (P < 0.05) are shown). Click here for file

Additional file 2: Table S1 List of identified proteins and their annotations from every analyzed 2D-gel protein spots. Click here for file
