# Supplementary material for: Litter size manipulation in laboratory mice: an example of how proteomic analysis can uncover new mechanisms underlying the cost of reproduction
Source: Front Zool. 2014 May 20;11:41. doi: 10.1186/1742-9994-11-41 (PMC4041047; doi:10.1186/1742-9994-11-41)
Supplement: Additional file 1: Figure S1 — Representative 2D-gel image of mouse liver proteins. Significantly different protein spots according to ANOVA analysis (P < 0.05) are shown). [file 1742-9994-11-41-S1.pptx]

## Slide 1
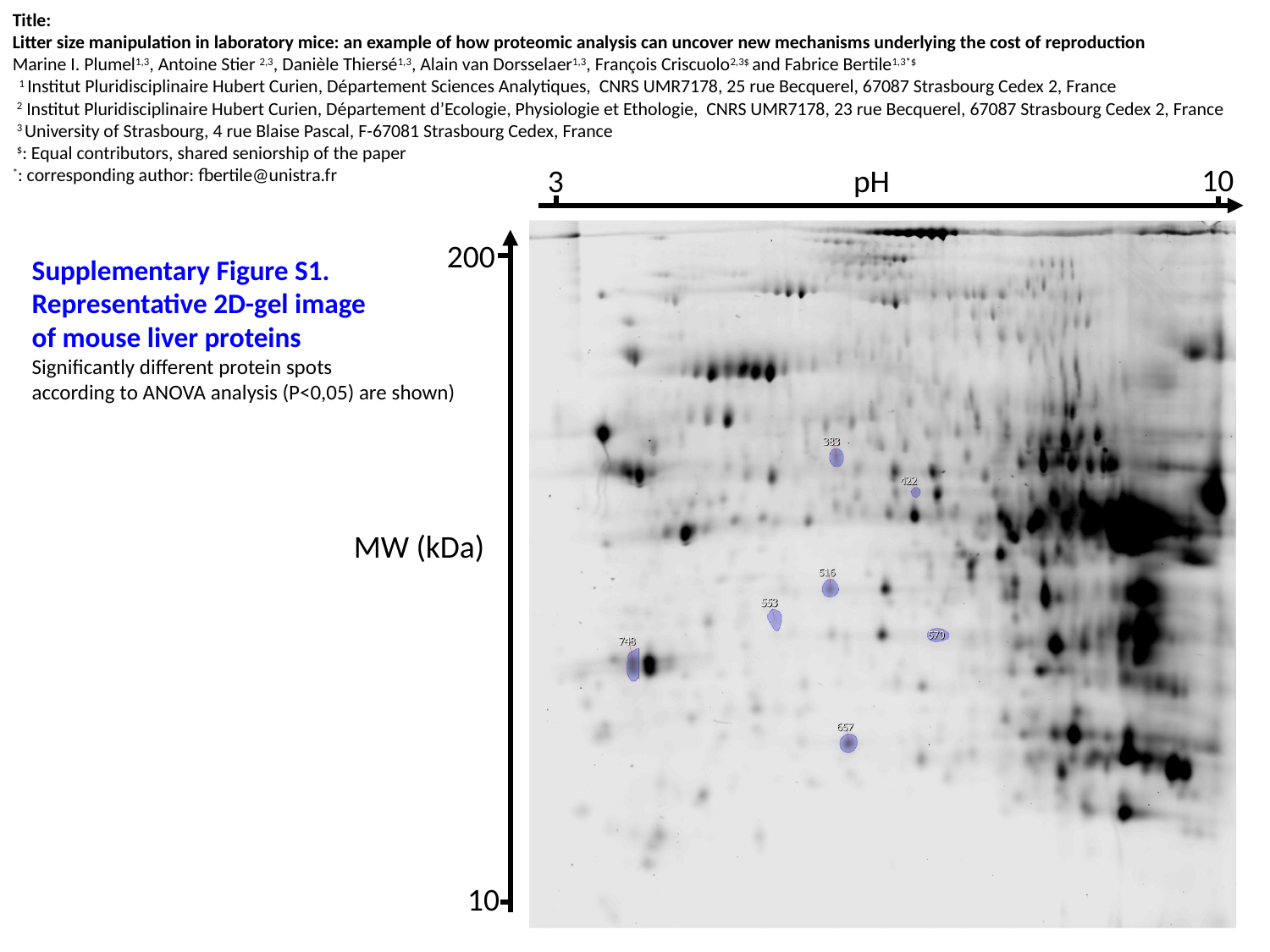

Title:
Litter size manipulation in laboratory mice: an example of how proteomic analysis can uncover new mechanisms underlying the cost of reproduction
Marine I. Plumel1,3, Antoine Stier 2,3, Danièle Thiersé1,3, Alain van Dorsselaer1,3, François Criscuolo2,3$ and Fabrice Bertile1,3*$
  1 Institut Pluridisciplinaire Hubert Curien, Département Sciences Analytiques, CNRS UMR7178, 25 rue Becquerel, 67087 Strasbourg Cedex 2, France
 2 Institut Pluridisciplinaire Hubert Curien, Département d’Ecologie, Physiologie et Ethologie, CNRS UMR7178, 23 rue Becquerel, 67087 Strasbourg Cedex 2, France
 3 University of Strasbourg, 4 rue Blaise Pascal, F-67081 Strasbourg Cedex, France
 $: Equal contributors, shared seniorship of the paper
*: corresponding author: fbertile@unistra.fr
10
3
pH
200
Supplementary Figure S1.
Representative 2D-gel image
of mouse liver proteins
Significantly different protein spots
according to ANOVA analysis (P<0,05) are shown)
MW (kDa)
10
